# Antimetastatic effect of nanodiamond-conjugated quercetin against colon cancer: an in vivo study

**DOI:** 10.55730/1300-0152.2704

**Published:** 2024-09-17

**Authors:** Firli Rahmah Primula DEWI, Sephia Tiara MARVIELLA, Sri Puji Astuti WAHYUNINGSIH, A’liyatur ROSYIDAH, Vuanghao LIM, Lionel Lian Aun IN, Amy Yi Hsan SAIK, Bimaji ARIYOGO, Mee Lee LOOI

**Affiliations:** 1 Department of Biology, Faculty of Science and Technology, Universitas Airlangga, Surabaya, Indonesia; 2 Research Center for Vaccine and Drug, National Research and Innovation Agency, Bogor, Indonesia; 3 Advanced Medical and Dental Institute, Universiti Sains Malaysia, Malaysia; 4 Department of Biotechnology, Faculty of Applied Sciences, University College Sedaya International, Kuala Lumpur, Malaysia; 5 Department of Pre-Clinical Sciences, M. Kandiah Faculty of Medicine and Health Sciences, Universiti Tunku Abdul Rahman, Malaysia; 6 Learning Planning and Development, Centre for Future Learning, Taylor’s University, Malaysia

**Keywords:** Anticancer, quercetin, nanodiamond, colon cancer, metastasis

## Abstract

**Background/aim:**

Quercetin (Q) is a compound that can inhibit the growth of cancer cells in the colon; however, to do so, a high dose is needed, requiring a drug delivery system to target cancer endothelial cells directly. This study investigates the potency of nanodiamond-conjugated quercetin (NDQ) as an anticancer drug against colon cancer in *Rattus norvegicus* induced by N-methyl N-Nitrosourea (MNU).

**Materials and methods:**

This study is experimental-based and was designed using a six-group treatment method, namely normal control (KN: not treated by MNU, nanodiamond (ND), or Q); negative control (K−: treated by MNU); positive control (K+: treated by MNU and capecitabine); ND (treated by MNU and NDs); Q (treated by MNU and Q); and NDQ (treated by MNU and NDQ). To induce colon cancer in rats, MNU (10 mg/Kg BW) was administrated intrarectally three times per week for four weeks. The treatment of Q (40 mg/Kg BW) or NDQ (40 mg/Kg BW) was given intraperitoneally twice a week for 6 weeks. Cancer progression of all cohorts was evaluated by performing body and colon weight measurements, which involved the following: ELISA assay-specific to metastatic marker matrix metalloprotein-9 (MMP-9), carcinoembryonic antigen (CEA), hypoxia-inducible factor 1 α (HIF1α), vascular endothelial growth factor, protein 53 (p53) and immunohistochemistry staining of Caspase-3 and Ki-67 proteins. Observation of cancer metastasis to the lung was also performed.

**Results:**

NDQ significantly inhibited cancer aggressiveness by causing an increment in body weight gain and the growth rate—while reducing the colon weight compared to the K− group. Moreover, decreased levels of MMP-9, CEA, HIF-1α, and Ki67 and increased levels of p53 and Caspase-3 were more significant in the NDQ group than in the Q group. The lung tumor metastases in the NDQ group were fewer than in the K− group.

**Conclusion:**

NDQ increased Q’s anticancer activity, suggesting that NDs have an effective drug delivery property.

## 1. Introduction

Colon cancer is the third-most diagnosed and second-most deadly form of cancer globally, according to GLOBOCAN 2018 data (Bray et al., 2018). This cancer is more often suffered by men than women and occurs three to four times more in developed than developing nations (Rawla et al., 2019). Colon cancer usually begins with a polyp and then grows gradually for 10–20 years before becoming an adenocarcinoma under unhealthy dietary and lifestyle conditions or due to hereditary or genetic modification (Watson and Collins, 2011; Rawla et al., 2019). The cancer’s malignant cells can invade nearby tissue and/or distant organs, such as the lung or liver, via the bloodstream (Valastyan and Weinberg, 2011; Rawla et al., 2019). These malignant cells can also invade the tissue’s capillary, proliferate to cause cancer, and invade the microenvironment in other organs, such as the lung or liver, called the metastases process (Quail and Joyce, 2013; Zhang et al., 2018). Treatments vary and have decreased mortality worldwide in the second and third categories mentioned previously despite increasing incidence rates. However, the 5-year survival rate for colon cancer is slightly lower, with 13% reported in stage IV (metastatic cancer) (Rawla et al., 2019). Hence, endeavors to obtain effective anticancer treatments for colon cancer are essential.

In accordance with the need for an effective anticancer regimen against colon cancer, the nanodiamond (ND) (a carbon-based nanomaterial) offers potency as a drug carrier due to its ability to impair drug efflux that leads to effective long-term drug accumulation in tumors (Horie et al., 2012; Gismondi et al., 2015; Behl et al., 2022). NDs can be designed to work with different functional groups, allowing interaction with water molecules or biologically relevant conjugates, which have promise in resolving drug resistance within the tumor microenvironment (Danhier et al., 2010; Behl et al., 2022). This interaction has resolved dose-dependent side effects by releasing the drug without any chemical modification, as reported in ND-conjugated anthracyclines (Zhu et al., 2012; Behl et al., 2022). However, the role of ND-loaded drugs in addressing metastasis has not yet been broadly elucidated.

Quercetin (Q), a flavonoid compound from the flavonol subclass, is prevalent in many fruits and vegetables, notably in the seeds of okra (*Abelmoschus esculentus* L.) (Khomsug et al., 2010; Reyes-Farias and Carrasco-Pozo, 2019). It is widely present in nature and the human diet and has powerful oxidative properties and diverse biological activities. Q’s covalent bonds provide cellular protection against oxidative stress, interacting with DNA through its hydroxyl groups to influence drug-DNA binding mechanisms (Song et al., 2020). Q enhances the body’s antioxidant capacity by regulating glutathione levels, scavenging reactive oxygen species (ROS), and modulating key signaling pathways like mitogen-activated protein kinase (MAPK), cytochrome C-type protein (NRFB), and adenosine monophosphate-activated protein kinase (AMPK) (Zhao et al., 2017; Gao et al., 2018; Qi et al., 2022). This compound also affects ROS-induced carcinogenesis, leading to genetic mutations, oncogene activation, and increased oxidative stress factors contributing to cancer development. A recent study showed that Q disrupts ROS metabolism, leading to apoptosis, by forming QC radicals (QC-O•) through peroxidase-catalyzed oxidation to scavenge harmful reactive peroxyl radicals (Biswas et al., 2022). Additionally, Q exhibits broad-spectrum antimicrobial activity, targeting a variety of gram-positive and gram-negative bacteria, fungi, and viruses. Its antimicrobial mechanisms include disrupting cell membranes, altering membrane permeability, inhibiting nucleic acid and protein synthesis, reducing virulence factor expression, inducing mitochondrial dysfunction, and preventing biofilm formation. This versatility makes Q a significant contributor to both antioxidant and antimicrobial defenses in the human body (Nguyen and Bhattacharya, 2022; Qi et al., 2022; Azeem et al., 2023).

Q also exhibits anticancer effects by inhibiting cell proliferation, promoting apoptosis, and altering cell-cycle progression. Several studies have shown that Q induces apoptosis and suppresses cell proliferation in various cancers, including colon, ovarian, breast, lung, oral, and prostate cancers (Hashemzaei et al., 2017; Hisaka et al., 2020; Azeem et al., 2023). The mechanisms for these effects involve several key cellular signaling pathways, including Wnt/β-catenin, phosphoinositide 3-kinase (PI3K)/protein kinase B (AKT), Janus kinase signal transducer and transcription activator, MAPK, protein 53 (p53), and nuclear factor kappa B (NF-κB). Moreover, Q targets specific tumor suppressors, oncogenic microRNAs, and long noncoding RNAs (Khan et al., 2016; Asgharian et al., 2022). Q has shown antimetastatic effects by disrupting uPA/uPAR functions and modulating key signaling pathways like NF-κB, PKC-δ, ERK1/2, and AMPKα (Li and Chen, 2018). However, Q’s therapeutic potential is limited by its low absorption, leading researchers to explore solutions for enhancing its bioavailability, such as incorporating it into nanoparticles or making structural chemical changes (Zang et al., 2021).

This study will elucidate the anticancer potential of nanodiamond–quercetin (NDQ) against N-methyl N-Nitrosourea (MNU)-induced colon cancer in *Rattus norvegicus*. This involves assessing tumor aggressiveness and measuring several cancer markers, including carcinoembryonic antigen (CEA), hypoxia-inducible factor 1 α (HIF1α), tumor suppressor p53, as well as the metastatic marker matrix metalloprotein-9 (MMP-9) and vascular endothelial growth factor (VEGF).

## 2. Materials and methods

### 2.1. Chemicals

ND carboxyl-modified (N1084, Tokyo Chemical Institute, Tokyo, Japan), Q (Q4951, Sigma-Aldrich, St. Louis, MO, USA), DMSO 25% (1.02952.1000, Sigma-Aldrich), N-(3-Dimethylaminopropyl)-N’-ethylcarbodiimide hydrochloride (EDC) (E7750, Sigma-Aldrich), N-hydroxysulfosuccinimide (Sulfo-NHS) (130672, Sigma-Aldrich), mPEG Amine (QBD10918, Sigma-Aldrich), MNU (N2939, Spectrum Chemical, New Brunswick, NJ, USA), Binecap (Ferron Par Pharmaceuticals, Surabaya, Indonesia), ELISA Kit for MMP-9 (SEKR-0027, Solarbio, Beijing, China), ELISA Kit for CEA (E1625Ra, BT Lab, Shanghai, China), ELISA Kit for VEGF (SEKR-0032, Solarbio), ELISA Kit for HIF1α (E0210Ra, BT Lab), and p53 (ER0394, FineTest, Boulder, CO, USA) were used in the experiments.

### 2.2. Conjugation of Q with NDs

The ND carboxyl-modified (2 mg/mL) was sonicated for 5 min, along with an EDC and Sulfo-NHS solution; 30 min after stirring, 200-μg mPEG-amine was added. The ND solution was centrifuged for 2 h at 12,000 rpm. The pellets were dispersed with 2.5-mM NaOH and sonicated for 5 min. Q (4 mg/mL) was dissolved in 25% DMSO, added to the ND solutions, and stirred overnight. The NDQ solution was centrifuged at 12,000 for 2 h. The pellets were collected and added to distilled water. The absorbance of Q before and after conjugation with NDs was measured using a microplate reader at 390 nm. To calculate the conjugation efficiency of the NDs with Q, the Q loading efficiency (QLE) was calculated using the following formula:


$$\text{QLE}=\frac{\text{compound added initially}-\text{the supernatant after centrifugation}}{\text{compound added initially}}\times 100$$

### 2.3. Characterization of the NDQ complex

The NDQ complex was characterized by transmission electron microscopy (TEM), UV-vis spectrophotometry, and Fourier transforms infrared (FT-IR) spectroscopy. The UV-vis measurement of Q before and after conjugation with the NDs was measured using a UV-vis spectrophotometer (Multiskan Go, Thermo Scientific, Waltham, MA, USA) at λ 200–800 nm with 1-nm intervals. The FT-IR spectroscopy measurements of Q, NDs, and the NDQ complex were performed using an FT-IR spectrometer (FT-IR Spectrometer Spectrum Two, PerkinElmer, Waltham, MA, USA) in the 4000–400 cm^−1^ range. The ND powders were placed in a diamond chamber, and the spectra were immediately recorded. For each sample, the signal obtained from the blank chamber was subtracted as a background. The spectral data were compared to those in the database to determine the functional groups in each sample.

### 2.4. Experimental design

All methods were carried out according to the relevant guidelines and regulations of the Animal Care and Use Committee of the Faculty of Animal Health, Universitas Airlangga (No. 2. KE.046.04.2021). All methods were reported in accordance with ARRIVE guidelines. Twenty-four female Wistar rats (*Rattus norvegicus*) were obtained from the Faculty of Veterinary Medicine of Universitas Airlangga, Indonesia. The rats were aged 4–6 weeks, with an average body weight of about 50–60 g, and were acclimatized for 2 weeks. The rats were observed and monitored daily by a trained research staff member. The rats were further divided into 6 groups: KN (normal control, the mice were not exposed to MNU, ND, and Q), K− (negative control, exposed to MNU 10 mg/Kg BW), K+ (positive control, exposed to MNU 10 mg/Kg BW + capecitabine 12.5 mg/Kg BW), ND (exposed to MNU 10 mg/Kg BW + ND 2 mg/Kg BW), Q (exposed to MNU 10 mg/Kg BW + Q 40 mg/Kg BW), and NDQ (exposed to MNU 10 mg/Kg BW + NDQ 40 mg/Kg BW). The dose of Q was determined according to a previous study by Oyewopo et al. (2021). The rats were induced with 10-mg/kg BW of MNU via intrarectal injection three times a week for 4 weeks to give rise to colon cancer (Roomi et al., 2005). The rats were treated with capecitabine, ND, Q, or NDQ twice a week for 6 weeks, according to the respective group. The animals were treated in a way that minimized animal suffering. The rats were then anesthetized using ketamine at a dose of 40 mg/kg per body weight via intramuscular injection. The serum was collected from the heart, and the aorta was cut before the organs were collected.

### 2.5. Cancer aggressiveness assessment and metastasis observation

To evaluate cancer aggressiveness, the research team measured the rat’s body and tumor weight and the growth rate. The measurement of the rat’s body weight was performed twice before both the MNU treatment and surgery. The calculation of body weight gain was carried out by subtracting the body weight before surgery from the initial body weight before treatment; calculating the growth rate was done using $\frac{Final\;body\;weight-Initial\;body\;weight}{Treatment\;period}$. Meanwhile, the colon weight was measured after surgery, and the rat colons were weighed using an analytical scale. Observation of metastases was performed by observing tumor formation within the lung. The number of tumor lumps was subsequently counted.

### 2.6. Analysis of cancer progression and metastasis

Analysis of cancer progression and metastasis was performed in blood serum and protein extracted from tumor tissue by ELISA assay-specific to MMP-9, CEA, HIF-1α, VEGF, and p53 and immunohistochemistry staining of Ki-67 and Caspase-3. Blood samples from the heart were centrifuged at 3000 rpm for 10 min to obtain serum. The serum was later stored at 20 °C. Approximately 0.4 g of colon tissue was ground using a mortar with 1 mL of PBS and 10 μL of PMSF. The homogenates were sonicated for 1 min and centrifuged at 5000 g for 5 min. The supernatant was collected and stored at −20 °C for later use. Standard solution, wash buffer, biotin-conjugate antibody solution, and Streptavidin-HRP solution were prepared by dilution according to product instructions. The standard and sample solutions were added to each well, incubated, the solution removed, and washed using a wash buffer. The biotin conjugate antibody was added to each well, incubated, the solution removed, and washed using wash buffer. Streptavidin HRP was added to each well, incubated, the solution removed, and washed using a wash buffer. Substrate solution was added to each well and incubated in a dark room. The solution was discarded and washed using a wash buffer. A stop solution was added to each well. The absorbance was measured using a microplate reader with a wavelength of 450 nm. The absorbance value was entered on the standard curve to determine the concentration of each biomarker.

Immunohistochemistry staining of Ki67 and Caspase-3 was performed according to the manufacturer’s protocols (IHC0007, FineTest, Boulder, CO, USA). Briefly, 4-μm thick paraffin-embedded tissue blocks were sectioned and deparaffinized. The slides were then blocked with blocking serum for 1 h and subsequently incubated with primary antibodies for Ki-67 (MA5-14520, Invitrogen, Waltham, MA, USA) and Caspase-3 (PA1-29157, Invitrogen) overnight at 4 °C. After being washed, the slides were incubated with a secondary antibody using poly-HRP goat antirabbit IgG. The color was developed using 3,3’-diaminobenzidine tetrahydrochloride for 10–15 min, and the sections were counterstained with hematoxylin. Images were acquired using a Nikon microscope (Tokyo, Japan) and digitally processed using ImageJ software to analyze the percentage area exhibiting positive staining of Ki-67 and Caspase-3.

### 2.7. Statistical analysis

The data was obtained in the form of average concentration ± SD. The Kolmogorov–Smirnoff test (p > 0.05) was used to determine data normality, followed by Levene’s test (p > 0.05) to assess data homogeneity. Furthermore, data were evaluated using one-way ANOVA (p > 0.05), and Tukey’s test was used to determine the significance between cohorts.

## 3. Results and discussion

### 3.1. Characterization of the NDQ complex

**Figure 1:**
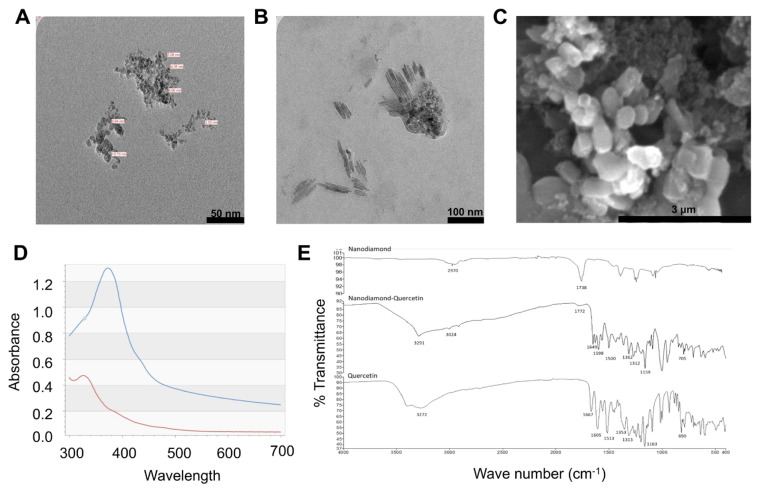
Characterization of quercetin conjugated onto nanodiamonds. (A) transmission electron microscopy visualization of nanodiamond particles; (B) transmission electron microscopy visualization of quercetin conjugated onto nanodiamonds; (C) SEM visualization of quercetin conjugated onto nanodiamonds at a magnification of 50,000×; (D) UV-vis spectrophotometer analysis of the quercetin before (blue) and after (red) conjugation with nanodiamonds; (E) Fourier transforms infrared spectroscopy analysis of NDs, quercetin, and quercetin conjugated onto nanodiamonds.

To characterize the NDQ complex, we performed visualization of ND and the complex using TEM and SEM imaging. According to the visualization, the diameter of ND alone was less than 10 nm, while the ND and Q complex sizes ranged from 130–500 nm (Figures 1A–1C). The UV-vis spectrometry analysis showed that the absorbance peak of Q before and after conjugation with ND was significantly reduced (Figure 1D). Q has two main absorption peaks: 240–280 nm and 340–440 nm (Buchweitz et al., 2016). According to the absorbance calculation in 390 nm, the Q-loading efficiency was 80.4%. To identify the interaction between Q and ND, the FT-IR of Q and ND were compared to those of the NDQ, as shown in Figure 1E. The IR spectrum of Q showed its characteristic bands (Catauro et al., 2015). In the Q spectra, OH stretching was observed at 3272 cm^−1^. At 1667 cm^−1^, the absorption of the C=O aryl ketonic stretch was detected. Stretch bands with C=C aromatic rings were observed at 1605 and 1513 cm^−1^. The OH bending of the phenol function was observed at 1353 cm^−1^. At 1313 cm^−1^, the band of the C-H bending in the aromatic hydrocarbon was observed, and the C-CO-C stretch and bending in ketone were observed at 1167 cm^−1^. Strong peaks for CH_3_ asymmetric stretching, C=O stretching, C-O stretching, and C-O stretching were seen in the NDs at 2970, 1738, 1365, and 1055 cm^−1^ (Petit and Puskar, 2018). The FT-IR spectra of the NDQ complex demonstrate the presence of the characteristic features of the corresponding Q spectra at 3291, 1649, 1598, 1500, 1362, 1312, and 1159 cm^−1^ and also ND spectra at 3024 and 1772 cm^−1^. It is possible that Q absorption into the NDs caused the wavenumber to shift. The FT-IR spectra of the NDQ complex showed characteristic bands of both Q and NDs, indicating that Q was successfully absorbed by the NDs.

### 3.2. NDQ increased the growth rate and p53 levels and reduced tumor weight in rats-induced colon cancer

**Figure 2:**
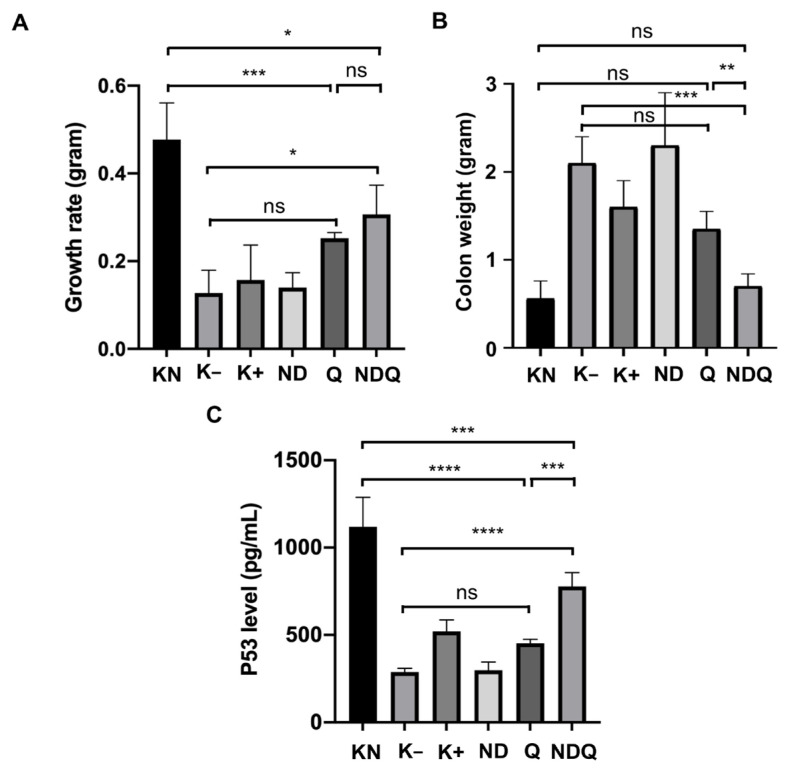
(A) Growth rate, (B) colon weight, and (C) p53 levels in rats-induced colon cancer following treatment with 40 mg/kg body weight of quercetin coupled with nanodiamonds for 6 weeks. Each bar represents mean ± SD (n = 4)

The calculation of the average growth rate and body weight gain of the rats was used to determine the aggressiveness of colon cancer formation. All groups experienced both weight gain and growth rate increments. In this regard, the improvement of growth rate in the K+ group (0.16 ± 0.08 g) and Q group (0.25 ± 0.01 g) were not significant compared to the K− group (0.13 ± 0.05 g). However, a significant increment compared to the K− group was observed in the NDQ group, in which the growth rate was 0.31 ± 0.06 g (Table; Figure 2A).

To investigate the effect of the NDQ complex on the proliferation of colon cancer cells, we measure the weight of tumor tissue in the colon. The tumor weight in the K−, K+, and ND groups was significantly higher compared to the KN group. The treatment of Q alone could not significantly reduce tumor weight. Colon weight in K+ (1.63 ± 0.30 g) and Q (1.35 ± 0.21 g) decreased but was not significant when compared to K− (2.12 ± 0.30 g). However, NDQ (0.83 ± 0.25 g) experienced a significant reduction in colon weight compared to K− (Figure 2B).

p53 is an important tumor suppressor that controls cancer development, including colon cancer. It is frequently deactivated in many human colorectal cancer (CRC) cases (Liebl and Hofmann, 2021). In this study, Q alone could not significantly increase the p53 level. Meanwhile, the level of p53 in NDQ (0.778 ± 0.078 ng/mL) was significantly increased compared to the K− group (0.288 ± 0.022 ng/mL) (Figure 2C).

### 3.3. NDQ increased Caspase-3 and reduced Ki-67 expression

**Figure 3:**
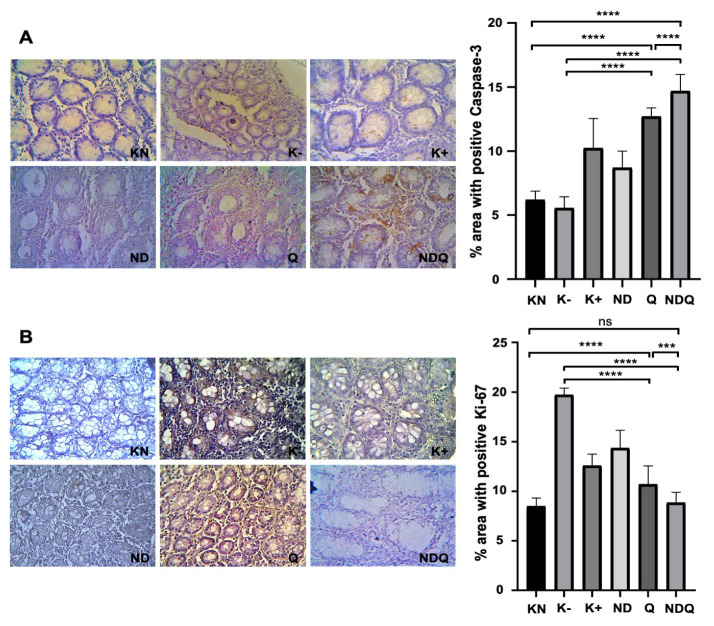
Percentage of area with positive Caspase-3 (A) and Ki-67 (B) in rats-induced colon cancer following treatment with 40 mg/kg body weight of quercetin coupled with nanodiamonds for 6 weeks. Mean values with different letters are significantly different at p < 0.05. Each bar represents mean ± SD (n = 4).

Caspase-3 and Ki-67 expression levels are critical determinants of tumor progression and metastasis, with Caspase-3 influencing apoptosis resistance and Ki-67 reflecting the proliferative potential of cancer cells (Xiao et al., 2013). The percentage of area with positive Caspase-3 staining in the Q (12.719 ± 0.676%) and NDQ groups (14.725 ± 1.291%) experienced a significant increase when compared to K− (5.555 ± 0.884%). Treatment with NDQ significantly increased the percentage of area with positive Caspase-3 compared to treatment with Q only (Figure 3A). In contrast, the percentage of area with positive Ki-67 in the Q (10.715 ± 1.861%) and NDQ groups (8.866 ± 1.043%) were significantly decreased compared to K− (19.730 ± 0.680%). The percentage of area with positive Ki-67 in the NDQ group was considerably lower than in the Q group (Figure 3B).

### 3.4. NDQ reduced the level of HIF-1α, VEGF, CEA, and MMP-9

**Figure 4:**
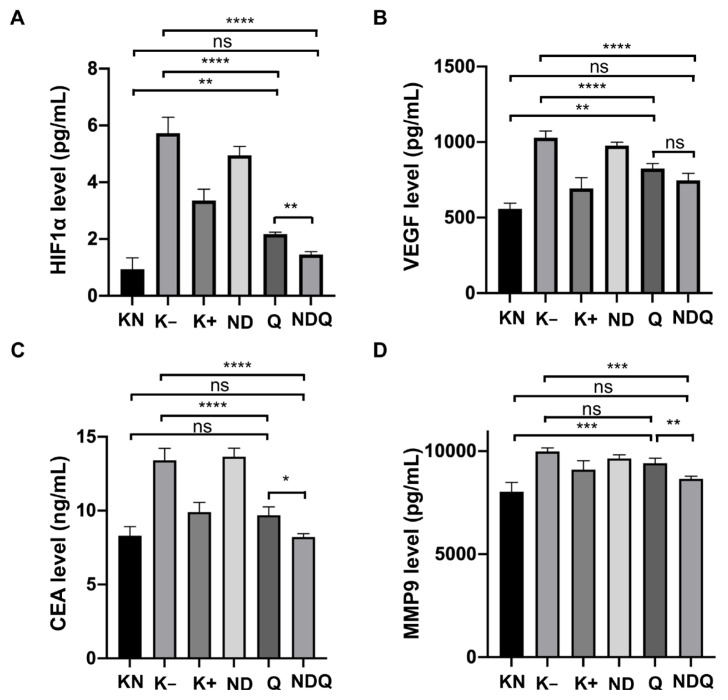
Levels of vascular endothelial growth factor (VEGF) (A), hypoxia-inducible factor 1 α (HIF-1 α) (B), carcinoembryonic antigen (CEA), (C) and metastatic marker matrix metalloprotein-9 (MMP-9) (D) in rats-induced colon cancer following treatment with 40 mg/kg body weight of quercetin coupled with nanodiamonds for 6 weeks. Mean values with different letters are significantly different at p < 0.05. Each bar represents mean ± SD (n = 4).

Metastasis occurs when cancer cells spread from the primary tumor site to surrounding tissues and distant organs and is the leading cause of cancer morbidity and mortality. Metastasis can be diagnosed by evaluating several biomarkers, including MMP-9, VEGF, and CEA (Cao et al., 2009; Huang, 2018; Alam et al., 2020). The HIF-1α transcription factor is a biomarker indicating an advanced stage and poor prognosis of CRC [23]. The level of HIF-1α in the Q (2.074 ± 0.221 ng/mL) and NDQ groups (1.333 ± 0.258 ng/mL) experienced a significant decrease when compared to K− (5.731 ± 0.561 ng/mL). Treatment with Q conjugated onto NDs significantly reduced the level of HIF-1α compared to treatment with Q only (Figure 4A). One of the target genes of HIF-1α is VEGF, a key protein during angiogenesis. The level of VEGF in the Q (840.37 ± 11.18 pg/mL) and NDQ groups (732.59 ± 43.64 pg/mL) significantly decreased when compared to K− (1029.17 ± 43.44 pg/mL). The level of VEGF in NDQ was lower than in the Q group. However, no significant difference was observed (Figure 4B).

CEA is one of the longest-used tumor markers for colorectal cancer (Sihotang and Putranto, 2022). Recently, a preoperative level of CEA >12 ng/mL was able to predict liver metastases of colorectal cancer at an accuracy rate of 85.3% (Alam et al., 2020). In the current study, the CEA level significantly decreased in the Q (9.542 ± 0.550 ng/mL) and NDQ groups (8.524 ± 0.632 ng/mL) compared to K− (13.410 ± 0.805 ng/mL). The level of CEA significantly diminished in the NDQ group compared to the Q group (Figure 4C). Another metastatic marker, MMP-9, was assessed in this study. The level of MMP-9 did not significantly decrease in the Q group (9.330 ± 0.262 ng/mL) compared to the K− group (9.988 ± 0.171 ng/mL). Interestingly, the NDQ group (8.851 ± 0.411 ng/mL) experienced a significant decrease compared to the K− cohort (Figure 4D).

### 3.5. NDQ prevented tumor metastasis to the lung

**Figure 5:**
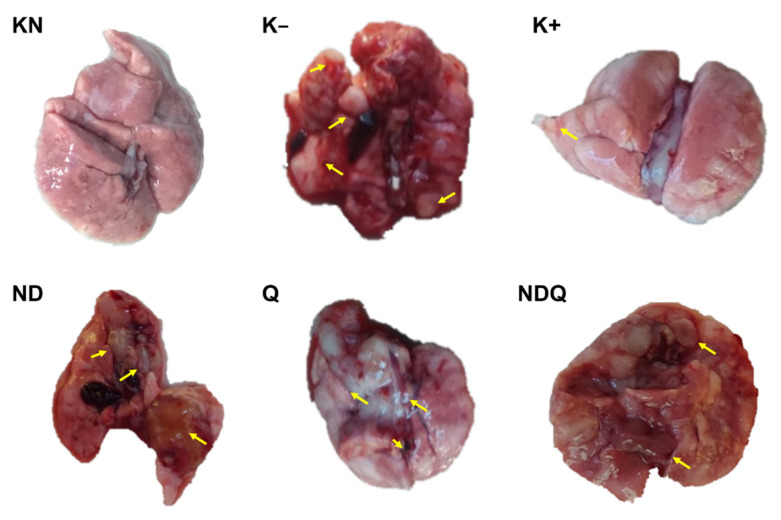
Tumor metastasis to the lung in rats-induced colon cancer following treatment with 40 mg/kg body weight of quercetin coupled with nanodiamonds for 6 weeks; yellow arrow = tumor lump.

Observations of metastases from colon cancer in this study were seen macroscopically in the lungs (Abou-Elkacem et al., 2013). Figure 5 shows the presence of tumor metastasis to the lung in all groups except for KN. The largest number of tumors was observed in the K− group. Compared to K−, the number of tumor lumps in the K+ and Q groups decreased, while the NDQ group experienced the most reduction of tumor numbers compared to other groups (Figure 5).

## 4. Discussion

Pathogenesis of colon cancer is highly convoluted because several factors, including sporadic, familial, and inherited factors, can cause induction. Among other causative factors, the sporadic factor is the most common cause of cancer, making up 70% of all total cases. Sporadic factors comprise environmental and dietary factors (Malki et al., 2020; Kandimalla et al., 2021). One carcinogenic agent is MNU. This compound acts as an alkylating agent that transfers an alkyl group and reacts with nucleophilic nitrogen and oxygen atoms in purine, pyrimidine, and the phosphate group, ending in a wide range of DNA adducts (Faustino-Rocha et al., 2015). Concerning the human diet, MNU can be found in many animal meats and processed food, often in high concentrations. The MNU-induced cancer model offers several advantages, such as reliable tumor induction and the ability to cause aggressive and locally invasive carcinoma in the mammary glands. Exposure to MNU intrarectally can lead to the development of cancer in the colon of *R. norvegicus* at an efficacy rate of 90% (Huang et al., 2020; Oyewopo et al., 2021). This efficient cancer induction was proved in a recent study where exposure to MNU 10 mg/kg BW intrarectally for 4 weeks caused colon tumor metastasis to the lung, demonstrated by the formation of tumor lumps.

Ki-67 and Caspase-3 expression analysis plays a pivotal role in understanding cancer progression and metastasis, offering valuable insights into the behavior of malignant cells (Li et al., 2022). Our study showed that the conjugation of Q onto NDs significantly increased the anticancer properties of Q by significantly reducing Ki-67 expression and increasing Caspase-3 expression. Ki-67, a proliferation marker, and its expression level provide valuable information about the rate of cell division in a tumor. High Ki-67 expression often correlates with aggressive tumor behavior, indicating a faster growth rate and increased risk of metastasis. Conversely, low Ki-67 expression is associated with slower growth and may suggest a more benign tumor. On the other hand, Caspase-3, an executioner caspase, is involved in apoptotic cell death. Its expression level is indicative of the apoptotic activity in a tumor. Reduced Caspase-3 expression may cause resistance to apoptosis, enabling tumor cells to evade cell death mechanisms, a hallmark of cancer progression (Xiao et al., 2013; Carneiro and El-Deiry, 2020).

Up to 25% of new cases of colorectal cancer have metastases at the time of diagnosis. Hepatic metastases are the most common cases, followed by pulmonary metastases. Colon cancer metastasis remains challenging in terms of cancer treatment since 50% of all colorectal cancer patients die from metastatic disease (Seyfried and Huysentruyt, 2013). Metastasis is characterized by the occurrence of epithelial–mesenchymal transition. A normal epithelial line, lined by a basement membrane, can proliferate locally, leading to adenoma. Further mutation gives rise to carcinoma in situ, which may fragment the basement membrane. In this case, invasive carcinoma cells further intravasate into lymp h or blood vessels, allowing their passive transport to distant organs (Huang et al., 2020). Colon cancer metastasis can be detected by MMP-9, VEGF, and CEA, while Ki-67, Caspase-3, HIF-1α, and p53 biomarkers help diagnose its malignancy.

Q, ubiquitously present in fruits and vegetables, is the primary representative of the flavonoid subclass of flavonols. It is known to have an anticancer effect due to its ability to reduce cell viability, promoting apoptosis and autophagy via its interference with PI3K/AKT/mTOR, Wnt/β-catenin, and MAPK/ERK1/2 pathways. In the event of metabolic programming, Q can inhibit PI3K/AKT/mTOR molecular pathways. The inhibition of PI3K/AKT decreases glucose uptake and glycolysis while inhibiting mTOR deactivating transcription factors such as HIF-1α. After a decrease in HIF-1α, VEGF is downregulated (Reyes-Farias and Carrasco-Pozo, 2019). A combination of Q and irinotecan (an anticolon cancer drug) reduces the concentration of angiogenesis-associated factors (VEGF-A and VEGF-receptor 2) (Lei et al., 2018). Deactivating the PI3K/AKT pathway by Q inhibits metalloproteinase (MMP) synthesis, promoting cell invasion (Huang et al., 2020). Consistently, Q decreased the protein levels of MMP-2 and MMP-9 in breast cancer cell lines (Jia et al., 2018). The reduction of HIF-1α, VEGF, and MMP-9 was demonstrated in this study. Q was also reported to increase p53 expression to induce apoptosis in U373MG malignant glioma cells (Kim et al., 2013). In another study, the MPAK pathway was downregulated, and the tumor suppressor gene Tp53 was upregulated in the distal colon mucosa of rats after being supplemented with Q (10 g/kg diet for 11 weeks) (Dihal et al., 2018). The increase of p53 was reported in the Q and NDQ groups after the rats were nourished with a Q 40 mg/kg BW diet twice a week for 6 weeks in our study. Furthermore, Q played a role in ameliorating the promotion of colon cancer by dimethyl-hydrazine in mice by reducing CEA levels in colon tissue plasma (Saleem et al., 2015).

The overall results of a recent study indicated a decrease in CEA, HIF-1α, MMP-9, and VEGF and an increase of p53 in the group treated by either capecitabine (K+), Q, or NDQ. However, only the NDQ group specifically showed a significant anticancer effect toward cancer progression compared to the K− (treated by MNU only) group. As previously mentioned, nanomaterials have become promising drugs for increasing efficacy and overcoming drug resistance. ND-conjugated drugs have improved the targeting of tumor cells and enhanced the potency of anticancer agents to reach maximum therapeutic effect (Garg et al., 2019). The effectivity of ND-conjugate in addressing chemotherapeutic resistance was shown in an in vivo study. The examination was performed in mouse models of liver and mammary cancer. ND-conjugated doxorubicin resolved drug efflux and significantly increased apoptosis and tumor growth inhibition beyond free doxorubicin treatment in both murine liver tumor and mammary carcinoma models (Chow et al., 2011). In another study, conjugation of NDs with paclitaxel, a microtubule inhibitor, and cetuximab, a specific monoclonal antibody against epidermal growth factor receptor (EGFR), enhanced the mitotic catastrophe and apoptosis induction of colorectal cancer cells. This conjugation also reduced tumor size in the xenograft EGFR-expressed human CRC tumors of nude mice (Lin et al., 2017). Differences in the cancer microenvironment exist, such as in normal tissue vascular endothelial cells that are compact and cancerous vascular endothelial cells loosely arranged, and these differences are useful for targeting cancer cells in nanocarrier research as a drug delivery method (Zhu et al., 2012).

## 5. Conclusions

ND-conjugated Q shows anticancer effects in rat colon cancer models induced by MNU. This drug formulation reduced disease aggressiveness by increasing the body weight and growth rate and decreasing the number of metastatic tumors in the lung. At the molecular level, NDQ lowers cancer biomarkers, CEA, and HIF-1α, as well as the VEGF and MMP-9 metastatic biomarkers. At the same time, the level of tumor suppressor p53 was found to have increased.

## Figures and Tables

**Table:** Average weight gain and growth rate ± SD of each experimental cohort.

Groups	Initial body weight ± SD (g)	Final body weight ± SD (g)	Weight gain ± SD (g)	Growth rate ± SD (g)
KN	83.00 ± 2.58	109.25 ± 2.22	26.25 ± 4.65	0.48 ± 0.08^a^
K−	83.75 ± 1.71	90.75 ± 1.26	7.00 ± 2.71	0.13 ± 0.05^cd^
K+	86.25 ± 2.22	95.00 ± 2.58	8.75 ± 4.19	0.16 ± 0.08^cd^
ND	83.75 ± 1.71	91.50 ± 1.29	7.75 ± 1.90	0.14 ± 0.03^cd^
Q	84.50 ± 2.08	98.50 ± 2.08	14.00 ± 0.82	0.25 ± 0.01^bc^
NDQ	84.00 ± 2.00	101.00 ± 2.65	17.00 ± 3.61	0.31 ± 0.06^b^

## Data Availability

All necessary data were presented in this manuscript.
